# Designing a Food Frequency Questionnaire for a Vegetarian Population in Germany by Means of Mixed-Integer Linear Programming

**DOI:** 10.3390/nu18101587

**Published:** 2026-05-16

**Authors:** Julia Blaurock, Thorsten Heuer, Kurt Gedrich

**Affiliations:** 1 Research Group Public Health Nutrition, ZIEL—Institute for Food & Health, Technical University of Munich, Weihenstephaner Berg 1, 85354 Freising, Germany; julia.blaurock@tum.de; 2 Department of Nutritional Behaviour, Max Rubner-Institut, Federal Research Institute of Nutrition and Food, Haid-und-Neu-Straße 9, 76131 Karlsruhe, Germany; thorsten.heuer@mri.bund.de

**Keywords:** vegetarian diet, food frequency questionnaire, mixed-integer linear programming, dietary assessment, nutritional epidemiology, proof of concept

## Abstract

Background: Food frequency questionnaires (FFQs) are important tools for dietary assessment in large epidemiological studies, playing a crucial role in evaluating the relationship between diet and health. However, adapting the food lists in FFQs to align with specific study objectives or target populations presents a considerable challenge. Methods: The present study develops a framework using mixed-integer linear programming (MILP) to minimize the food list of an FFQ using a vegetarian population in Germany as a proof of concept. Constraints of the optimization ensured that the selected food items have a certain nutrient coverage and variance coverage, as well as an appropriate aggregation level. Nutrient intake for three scenarios for FFQs was compared with 24 h recalls (24HR) using R^2^, calculated through linear regression. The three scenarios were: 1. FFQ reflecting the effect of categorizing portion sizes, 2. FFQ reflecting the effect of selecting food items, 3. FFQ reflecting the effect of categorizing portion sizes and selecting food items. Results: Length of minimized FFQs increased with a higher proportion of nutrient coverage and variance coverage. Including aggregation of food items produced shorter FFQs than FFQs that only contain food items at a lower aggregation level. R^2^ values across the three scenarios showed that the FFQ captured most of the between-person variation in nutrient intake that was observed in the 24HR. Conclusions: MILP offers a reliable and data-driven framework for compiling optimized FFQs.

## 1. Introduction

Vegetarian diets have gained popularity globally in the last few years and are associated with ethical considerations and with human and planetary health benefits. The number of vegetarians in Germany has increased in recent years. According to the 2023 Nutrition Report in Germany, 46% of respondents followed a flexitarian diet, meaning they occasionally avoid meat consumption. A total of 8% of the respondents follow a vegetarian diet and 2% a vegan diet [1]. To accurately assess vegetarian diets, dietary assessment tools like food frequency questionnaires need to reflect specific characteristics in vegetarian diets that differ from omnivorous diets [2].

Food frequency questionnaires are commonly used in nutritional epidemiology studies. Depending on the study aim and study participants, FFQs assess habitual dietary intake with a predefined food list. As dietary habits change with specific diets or certain populations, food lists must be adapted to those changes [3,4].

Besides the predefined food list, FFQs comprise questions about portion sizes and consumption frequencies [3].

Portion sizes can be measured differently in FFQs. Semi-quantitative FFQs collect data on portion sizes using standard sizes [3]. Standard portion sizes are calculated with, e.g., median or mean values derived from underlying dietary data like 24 h dietary recalls [5]. Quantitative FFQs include the selection of different portion sizes, e.g., the estimation of portion sizes in household measures or grams or the selection of portion size photographs [3,6,7]. Portion sizes for these photographs can be based on percentiles from an underlying dietary intake dataset, e.g., 24HR [7].

Vegetarians’ dietary pattern differs from omnivores with potentially lower intakes of certain essential nutrients, such as omega-3 polyunsaturated fatty acids, iron, zinc, calcium, riboflavin, and cobalamin [2]. Compared to omnivores, vegetarians exclude a wide range of animal products from their diet while they consume higher amounts of foods like seeds, tofu, and other meat substitutes [8,9]. If these products are not adequately represented in FFQs, it could result in inaccurate dietary assessments among vegetarians.

Against this background, there is a need for a tailored FFQ that accurately reflects the dietary habits of the vegetarian population in Germany. To ensure that an FFQ is accurate, the food list needs to include all food items that contribute substantially to nutrient intakes and explain a high percentage of the variance in nutrient intakes in order to reflect differences in dietary intake between persons. At the same time, food lists should not be unnecessarily long to minimize the burden of response [10].

An essential factor in shortening the food list is the aggregation of food items. However, aggregation of food items also influences the accuracy of an FFQ. When food items are aggregated, different single food items with varying nutritional profiles are combined into one aggregated item in the FFQ. The nutrient content of an aggregated item typically represents a weighted average of the nutritional profile of all food items included to accurately reflect the dietary habits of the study population [11,12].

This paper designs an optimized FFQ for vegetarians using mixed-integer linear programming (MILP) optimization techniques. The objective of the optimization is to minimize the number of food items while selecting food items with large nutrient coverages and large in-between subject variations. Details about the methodology are presented elsewhere [13,14].

The paper pursues the following objectives:

1. To develop an optimization framework for FFQs by means of MILP, applied to a vegetarian population in Germany.
2. To calculate portion sizes for the optimized FFQs and to estimate dietary intake based on three different scenarios for FFQs:
  - FFQ with
    (a) All food items that were consumed in the 24HR.
    (b) Calculated portion sizes (percentiles from the 24HR).
  - Optimized FFQ with
    (a) Minimized number of food items.
    (b) Original portion sizes from the 24HR.
  - Optimized FFQ with
    (a) Minimized number of food items.
    (b) Calculated portion sizes (percentiles from the 24HR).
3. To compare R^2^ from the three different scenarios for FFQs.

## 2. Materials and Methods

### 2.1. Study Population

The development of an optimized FFQ is based on data from the most recent German National Nutrition Survey (NVS II). Food consumption data were collected between 2005 and 2007 from 13,926 participants aged 14 to 80 years [15]. Among them, 288 participants were identified as vegetarians (208 women and 80 men; mean age 41 years) and formed the basis for the present analysis. All dietary data used in this study stem from this survey period.

### 2.2. Dietary Assessment

Dietary data was collected through two 24 h recalls (24HR) using EPIC-Soft [16]. In computer-aided telephone interviews, the respondents initially provided a general overview of their food and beverage intakes on the previous day, which were subsequently described in more detail. Estimation of portion sizes was supported using photos, household measures, and standard units [16,17]. Dietary intake data for each person was calculated as an average of the two 24HR.

In EPIC-Soft, every consumed item was categorized into food items or recipes (including recipe ingredients). Items categorized as food items were directly linked with a seven-digit code from the German Nutrient Database (BLS), version 3.02 [18]. A total of 183 recipes had been reported, which were linked to their corresponding BLS recipe codes.

For the aggregation of food items, the hierarchical structure of the BLS was used, classifying food items based on a 7-digit system. The first digit consists of a letter distinguishing 20 main food groups, e.g., main group B classifies food items as bread and rolls. The second digit indicates subgroups within those main groups. For example, in the case of bread and rolls, this could be wholemeal breads or white breads [19]. Each subgroup is assigned a unique digit. The third and fourth digits identify specific food items within the subgroups. In the case of wholemeal breads, this could be, e.g., wholemeal wheat bread.

Based on this structure, two aggregation levels were defined for the present paper: For the first aggregation level, food items were classified according to the first two digits of the BLS code. For the second aggregation level, food items were identified according to the first three BLS digits. Each food item reported by vegetarian subjects was ultimately described based on these two aggregation levels (*N* = 123 food items on aggregation level 1 and *M* = 421 food items on aggregation level 2).

### 2.3. Optimization with MILP

Given a set of foods *N* and *M*, respectively, and a set of nutrients *J*, the optimization problem can be formulated as follows [14]:
$\left(1\right)\;\;minimize\;\;\sum_{n=1}^{N}x_{n\;}\;\;+\sum_{m=1}^{M}x_{m\;}\;\;\;\;\;\;\;$$\mathrm{for}\;x_{n}$ ∈ {0,1} and $x_{m}$ ∈ {0,1} *n* = specific food item at aggregation level 1 *m* = specific food item at aggregation level 2 for *n* ∈ {1, …, N} for aggregation level 1 with *N* = 123 and *m* ∈ {1, …, M} for aggregation level 2 with *M* = 421 $x_{n}$ is a binary decision variable indicating whether food item *n* is in the food list $\mathrm{if}\;x_{n}$ = 1, food item *n* is included, if $x_{n}$ = 0, food item *n* is not included $x_{m}$ is a binary decision variable indicating whether food item *m* is in the food list $\mathrm{if}\;x_{m}$ = 1, food item *m* is included, if $x_{m}$ = 0, food item *m* is not included$(2)\;\;\;s.t.\;\;\;\sum_{n=1}^{N}x_{n}\;\cdot \;C_{1,n}+\sum_{m=1}^{M}x_{m}\;\cdot \;C_{1,m}\;\geq \;\;b$ ⋮ $\sum_{n=1}^{N}x_{n}\;\cdot \;C_{j,n}+\sum_{m=1}^{M}x_{m}\;\cdot \;C_{j,m}\;\geq \;\;b$ ⋮ $\sum_{n=1}^{N}x_{n}\;\cdot \;C_{41,n}+\sum_{m=1}^{M}x_{m}\;\cdot \;C_{41,m}\;\geq \;\;b$for *j* ∈ {1, …, 41} *j* = specific nutrient $C_{j,n}$ = percentual contribution of food item *n* to the overall intake of nutrient *j* $C_{j,m}$ = percentual contribution of food item *m* to the overall intake of nutrient *j*
$C_{j,n}=\;\frac{\;q_{j,n}}{\sum_{n=1}^{N}q_{j,n}}$
$C_{j,m}=\;\frac{\;q_{j,m}}{\sum_{m=1}^{M}q_{j,m}}$ $q_{j,n}=$ intake of nutrient *j* from food item *n* over all individuals *i* = 1, …, 288 $q_{j,m}=$ intake of nutrient *j* from food item *m* over all individuals *i* = 1, …, 288
b = arbitrary threshold, where b ∈ [0,1] with *b* ∈ {0.80, 0.85, 0.90, 0.95} in this study$(3)\;\;\;\;\;\;\;\;\;\;\;\sum_{n=1}^{N}x_{n}\;\cdot \;S_{1,n}\;+\;\sum_{m=1}^{M}x_{m}\;\cdot \;S_{1,m}\;\geq \;b$ ⋮ $\sum_{n=1}^{N}x_{n}\;\cdot \;{\;S}_{j,n}\;+\;\sum_{m=1}^{M}x_{m}\;\cdot \;S_{j,m}\;\geq \;b$ ⋮ $\sum_{n=1}^{N}x_{n}\;\cdot \;{\;S}_{41,n}\;+\;\sum_{m=1}^{M}x_{m}\;\cdot \;S_{41,m}\;\geq \;b$$S_{j,n}$ = percentual contribution of food item *n* to the sum of variances of the overall intake of nutrient *j* $S_{j,m}$ = percentual contribution of food item *m* to the sum of variances of the overall intake of nutrient *j* $S_{j,n}=\;\frac{Var(q_{j,n})}{\sum_{m=1}^{M}Var(q_{j,m})}$ $S_{j,m}=\;\frac{Var(q_{j,m})}{\sum_{m=1}^{M}Var(q_{j,m})}$ $Var(q_{j,n})=\frac{1}{I}\sum_{i}^{I=288}{\left(q_{j,n,i}-\;\bar{q_{j,n}}\right)}^{2}$ $Var(q_{j,m})=\frac{1}{I}\sum_{i}^{I=288}{\left(q_{j,m,i}-\;\bar{q_{j,m}}\right)}^{2}$ for *i* ∈ {1, …, I} *i* = individuals with *I* = 288 total number of individuals
$q_{j,n,i}=$ intake of nutrient *j* from food item *n* by person *i*
$q_{j,m,i}=$ intake of nutrient *j* from food item *m* by person *i*$(4)\;\;x_{n}+\;x_{m}\leq \;1$ ∀n∈ {1, …, N}, ∀m ∈ G(n)*G*(*n*) = set of food items at lower aggregation level corresponding to *n*

The objective of the optimization is to minimize the number of food items (formula 1).

The first set of constraints (formula 2) reflects nutrient coverage. They ensure that those food items are selected that contribute substantially to the intake of specific nutrients.

The second set of constraints (formula 3) refers to variance coverage. They ensure that those food items are selected that contribute substantially to the interindividual variance in the intake of specific nutrients. A food item that contributes substantially to the mean nutrient intake of the population does not necessarily explain differences in nutrient intake between individuals, and vice versa. Therefore, both nutrient coverage and variance coverage constraints are needed to ensure that the FFQ accurately reflects dietary intake and is capable of ranking individuals by their nutrient intake. A more detailed explanation of the optimization with MILP can be found elsewhere [13,14].

The optimization method described by Blaurock et al. has been extended by allowing different aggregation levels [14]. The third set of constraints (formula 4) is the duplication constraints. They ensure overlapping food items at aggregation levels 1 and 2 are not chosen simultaneously.

In the following, the mathematical model is described in detail.

For nutrient coverage constraints (formula 2), the percentage contribution of food items to nutrient intake was calculated for aggregation levels 1 and 2, respectively. Thus, the percentage contribution of nutrient coverage for each food item at each aggregation level was computed.

It was assumed that the FFQs with aggregated food items provided nutrient values that are weighted means derived from representative dietary intake data of a population. Therefore, the average nutrient intake is independent of the aggregation level in the FFQ. Consequently, to calculate the percentage contribution of each food item for each of the two aggregation levels, the same absolute nutrient intake for FFQs with aggregation level 1 and aggregation level 2 was assumed.

As food items at aggregation level 1 are more aggregated, the percentage contribution of the total nutrient intake from food items at aggregation level 1 is higher than that of the food items at aggregation level 2. For example, wholemeal breads (aggregation level 1) explain 1.2% of the total energy intake. In comparison, the more specific food item, wholemeal wheat bread (aggregation level 2), explains only 0.06% of the total energy intake.

Nutrient variance constraints (formula 3) aim to model R^2^. For nutrient variance constraints, the percentage contribution of food items to the sum of nutrient variances was calculated for each food item of the two aggregation levels. Thus, the percentage contribution of variance coverage for each food item at each aggregation level was computed.

When food items are aggregated into broader categories at aggregation level 1, there is a reduction in the variability of nutrient intake compared to food items at aggregation level 2. Thus, the overall sum of nutrient variances is lower on aggregation level 1 than on aggregation level 2. With its less aggregated food items, aggregation level 2 provides more information on variance than aggregation level 1. The larger sum of variance $\sum_{m=1}^{M=421}Var(q_{j,m})$ observed at aggregation level 2 was used as the denominator to determine percentage contributions to the sum of variance (see formula 3).

As food items at aggregation level 1 are more aggregated, the contribution to nutrient variance is, on average, lower than the total contribution of the less aggregated food items at aggregation level 2. In the present study, the sum of variance over all food items for energy intake at aggregation level 1 is approximately one-third smaller than the sum of variance at aggregation level 2.

Since food items at aggregation level 2 are full subsets of those at aggregation level 1, each food item must be included only once in the analysis, either as part of aggregation level 1 or 2. To achieve this, a duplication constraint (formula 4) was formulated pairwise to prevent the simultaneous selection of a food item at the higher aggregation level 1 and any of its corresponding sub-food items at the lower aggregation level 2. At the same time, the model still allows the selection of multiple food items at the lower aggregation level 2. For example, both *wholemeal bread* and *wholemeal wheat bread* cannot be selected together as *wholemeal wheat bread* (aggregation level 2) is a subset of *wholemeal bread* (aggregation level 1).

Additionally, an optimization that only includes aggregation level 2 was performed and compared with the optimized FFQs that select between two aggregation levels (aggregation levels 1 and 2).

MILP analyses were performed for b ∈ {0.80, 0.85, 0.90, 0.95}.

Calculations were performed in R, version 4.3.3. For the optimization, the ROI package, version 1.0-1, was used.

### 2.4. Performance Evaluation

In general, dietary intake data from 24HR and from FFQ differ in terms of identification of food items and quantification of intake. Whereas 24HR are highly flexible and accept any information provided by subjects, FFQs require the categorization of the consumed food items and their consumed quantities (based on predefined portion sizes and consumption frequencies).

To evaluate the methodological performance of the optimized FFQs under controlled conditions, the 24HR data from vegetarian subjects were categorized as if the data were collected by the FFQ. The necessary categories of food quantities were defined based on percentiles of the reported food quantities in the 24HR data. For each FFQ item, seven different portion sizes with equal intervals were computed, ranging from XS to XL. These portion sizes were based on the average values derived from the two 24HR reported by each participant.

To assess how well the optimized FFQs reflect dietary intake, linear regression models were fitted with nutrient intake from mimicked FFQs as the independent variable and the original 24HR intake as the dependent variable.

Three different scenarios to estimate nutrient intakes from the FFQ were assessed to isolate distinct sources of information loss that arise from the FFQ design.

1. Effect of categorizing food quantities
  FFQ with
    (a) All food items that were consumed in the 24HR.
    (b) Calculated portion sizes (percentiles from the 24HR).

For this scenario, no food items were excluded by the optimization MILP selection. Portion sizes were calculated based on percentiles of portion sizes from 24HR from the NVS II. The differences in nutrient intake between the 24HR and the FFQ stem from different portion sizes that were used in the two methods.

2. Effect of selecting food items
  Optimized FFQ with
    (a) Minimized number of food items.
    (b) Original portion sizes from the 24HR.

For this scenario, reported food items in the 24HR were matched to the optimized food list based on b = 0.80 and all 41 nutrients considered (i.e., J = 41). The respective food quantities were taken from the 24HR without further modification.

3. Effect of selecting food items and categorizing food quantities
  Optimized FFQ with
    (a) Minimized number of food items.
    (b) Calculated portion sizes (percentiles from the 24HR).

This scenario combines the two scenarios above: Reported food items in the 24HR were matched to the optimized food list (*b* = 0.80 and *J* = 41), and the consumed quantities were categorized according to the seven portion sizes mentioned above.

R^2^ quantifies how much of the variation in dietary intake between individuals observed in 24HR can be explained by the FFQs. Thus, R^2^ is used as a performance metric to assess how strongly the FFQs reproduce the between-person variation observed in the 24HR. However, R^2^ alone may not adequately capture the uncertainty associated with the regression model. To quantify the uncertainty associated with R^2^, 95% confidence intervals were calculated with bootstrapping [20,21]. Bootstrapping is a resampling technique that repeatedly draws samples with replacement from the observed data and calculates R^2^ for each bootstrap sample. The number of bootstrap repetitions for the present calculation was set to 1000.

## 3. Results

### 3.1. Optimization

FFQs were created with MILP for different thresholds *b* (0.80; 0.85; 0.90; 0.95) and for different nutrients of interest: first, for energy intake only, and second, for 41 nutrients, including macronutrients, vitamins, minerals, and trace elements (see Table A1 in Appendix A).

Table 1 shows the number of food items included in the FFQ for energy intake and 41 nutrients when two aggregation levels (aggregation level 1 and aggregation level 2) are considered. For energy intake, the number of included food items ranged from 44 (for *b* = 0.80) to 76 (for *b* = 0.95). For all 41 nutrients, the number of food items ranged from 66 (for *b* = 0.80) to 105 (for *b* = 0.95). The total number of food items increased with higher *b* and when more nutrients were considered.

The percentage of food items included at aggregation level 2 increased with the number of included nutrients: When looking only at energy intake, the percentage of food items included at aggregation level 2 varied from 6% (for b = 0.85) to 11.8% (for b = 0.95). However, considering all 41 nutrients, this percentage increased from 21.3% (b = 0.85) to 25.3% (*b* = 0.90).

Table 2 shows the number of food items included in the FFQ when only aggregation level 2 is considered. Optimized FFQs contained, on average, 2.5 times as many food items as FFQs that considered both aggregation levels. For example, for *b* = 0.80 and energy intake, the FFQ considering aggregation levels 1 and 2 contains 44 food items, whereas the FFQ considering only aggregation level 2 includes 114 food items.

### 3.2. Calculation of Nutrient Intakes and R^2^ for FFQs

**Figure 1:**
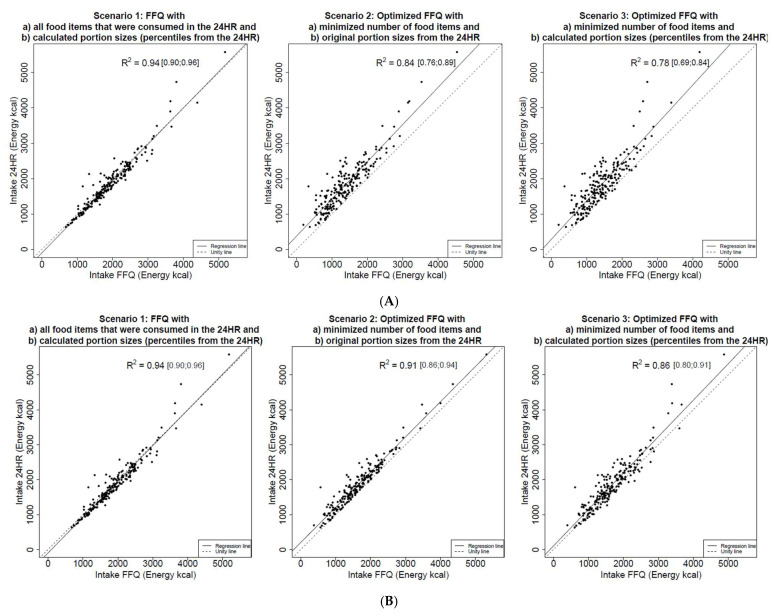
(**A**). Linear regression models for energy intake with three different scenarios to compose the FFQ with threshold *b* = 0.80; dots represent the overall energy intake of individuals. The dashed line represents the linear regression line, and the dotted line represents the line of identity. The R^2^ values are presented with 95% bootstrap confidence intervals. (**B**). Linear regression models for energy intake with three different scenarios to compose the FFQ for threshold *b* = 0.90; dots represent the overall energy intake of individuals. The dashed line represents the linear regression line, and the dotted line represents the line of identity. The R^2^ values are presented with 95% bootstrap confidence intervals.

The agreement between dietary intake assessed by FFQs and 24HR was assessed with R^2^ values from linear regression models. The R^2^ values and their 95% confidence intervals were calculated for three different FFQ scenarios across 41 nutrients. The detailed results are presented in Table A2 in Appendix A. For illustration, Figure 1 shows the linear regression models for the three different scenarios. In each plot, the dots represent the total amount of energy intake of each individual. In the first scenario, differences between the FFQ and the 24HR were caused by the categorization of food quantities. Mean value for the coefficient of determination was R^2^ = 0.94, the highest among the three scenarios, with values ranging from R^2^ = 0.87 (CI: 0.73;0.97) to R^2^ = 0.98 (CI: 0.96;0.99), indicating a strong agreement between the FFQ and the 24HR. In the second scenario, differences between the FFQ and the 24HR were caused by the selection of food items in the FFQ. Coefficients of determination were generally slightly lower than in the first scenario, ranging from R^2^ = 0.76 (CI: 0.51;0.88) to R^2^ = 0.98 (CI: 0.97;0.99), with a mean value of R^2^ = 0.86. In the third scenario, differences between the FFQ and the 24HR were caused by the categorization of portion sizes and the selection of food items. This scenario yielded the lowest mean value of the coefficient of determination with R^2^ = 0.81, with values ranging from R^2^ = 0.60 (CI: 0.38;0.70) to R^2^ = 0.94 (CI: 0.87;0.97).

## 4. Discussion

The study developed FFQs for a vegetarian population with MILP by selecting the smallest number of food items and their respective aggregation level while adhering to the specified constraints. Subsequently, portion sizes for food items were calculated from percentiles of dietary intake data from the NVS II. After that, the methodological performance of the optimized FFQs was evaluated using 24HR data from the NVS II. The R^2^ values from the optimized FFQs were calculated and compared for different nutrients.

### 4.1. Data

Compared to an optimized FFQ for omnivores, the food list for the vegetarian FFQ is shorter [14]. There are two main reasons for this: Firstly, the vegetarian population is a subset of the overall population of the NVSII for which the comparative FFQ was developed. This smaller subgroup consumed a smaller variety of foods than the large group. Secondly, dietary intake data was assessed in 2005–2007 when fewer vegetarian and vegan substitute products were available. With the growing number of vegetarians and vegans, the market for such products has also expanded. Over the last few years, the daily consumption of vegetarian and vegan alternatives to animal products has grown worldwide [22]. In Germany, the proportion of people consuming vegetarian and vegan alternatives on a daily basis increased from 5% in 2020 to 10% in 2023. This increase is particularly evident among people under the age of 44 [1].

Due to the lack of more recent consumption data in Germany, the variety of vegetarian and vegan products currently available is not yet reflected in the vegetarian FFQs that were created. However, this paper provides a versatile framework that can easily be applied to more recent dietary intake data when available.

To allow for a more straightforward and interpretable analysis, this study assumed that food items are uncorrelated. However, it is common for certain food items to be consumed together, such as in meal combinations [23]. Consequently, the correlation between food items might lead to inaccuracies in their contribution to the variance. In particular, food items that are frequently consumed together might have their individual variance contributions overestimated.

To calculate nutrient intakes from the FFQ, nutrient values of the aggregated food items were weighted with food intakes from the NVSII. As the weighted nutrient values were, in turn, applied to the same population again, in this case, aggregation of food items did not affect the accuracy of nutrient estimation in the FFQ. However, where this assumption does not apply, aggregation of food items can affect the accuracy of nutrient estimation in the FFQ. Therefore, it is important that weighted nutrient values represent the populations’ dietary intake [12,24].

### 4.2. Number of Food Items

The length of food lists for optimized FFQs varied depending on the threshold values *b* and the number of nutrients included. The number of food items in an FFQ impacts its accuracy [25]. Short FFQs are associated with higher response rates, while long food lists could lead to overestimation. Therefore, Tabacchi et al. suggested that a food list should not be longer than 114 food items [25]. These findings are in accordance with the length of the optimized FFQs, where the longest optimized FFQ contains 105 food items, which explains 95% of nutrient coverage and nutrient variance of 41 nutrients. Longer FFQs (more than 200 food items) might be better at ranking people than shorter FFQs (around 100 food items) [26]. However, the constraints in the optimization ensure that, depending on the threshold value *b*, at least 80%, 85%, 90%, or 95% of the variance is explained by the included food items. This ensures sufficient ranking accuracy despite minimizing the food list.

An option for shortening food lists can be the aggregation of food items [12].

When comparing the length of the food lists that allow two aggregation levels to those that allow only the lower aggregation level (aggregation level 2), it is consistently observed that the food lists with two aggregation levels are shorter. This result strongly supports that aggregation of food items should be applied when developing FFQs, as it produces shorter food lists.

Not only the number of overall food items but also those at aggregation level 2 increases with both a larger threshold *b* and the number of nutrients included.

One reason for a higher proportion of food items at aggregation level 2 is associated with a higher threshold *b*. The second set of constraints states that the sum of all the proportions of the included food items must achieve a minimum of 80%, 85%, 90%, or 95% of the total nutrient variances. However, selecting food items solely at aggregation level 1 would account for only 65% of the total variances. Therefore, as the threshold *b* increases, it becomes necessary to include food items at aggregation level 2. This is because the proportion of nutrient variance for food items at aggregation levels 1 and 2 is based on the total variance at aggregation level 2. The sum of variance of aggregation level 2 was chosen as the overall variance, as in the present study, food items were limited to be described at aggregation level 2. Consequently, all the variance in the data is attributed to food items at aggregation level 2. As a result, to fully explain nutrient variance in the data, it is necessary to include food items at aggregation level 2.

The other reason for a higher proportion of food items at aggregation level 2 is associated with more nutrients considered in the optimization. For some nutrients, variability between persons can be attributed to a few specific food items. For example, nutrients like vitamin A, beta carotene, or alcohol require only a few food items to substantially explain between-person variance [27].

Selecting a higher number of less aggregated food items may result in a longer FFQ, but it enhances the explanation of variance between individuals, especially for nutrients that are mainly found in some particular food items [28,29]. Conversely, higher aggregated food items could cause measurement errors as nutrient intake of specific food items that explain between-person variance could be inaccurate [12].

### 4.3. Aggregation of Food Items

Aggregating food items in an FFQ is often a trade-off between making the FFQ shorter and easier to complete on the one hand and losing information due to less-detailed information about nutrient coverage and variance coverage on the other hand [30].

The level of detail in the food items included in the optimization influences the granularity of the generated FFQs. In this study, the first three digits of the BLS classification system are considered as the most detailed categorization of food items possible, as they identify single food items and offer substantial detail [31].

In the present study, food items were aggregated according to the hierarchical system of the BLS. Consequently, aggregation in the FFQs resulting from optimization agrees with the BLS aggregation.

However, there are also other approaches to aggregate food items.

These methods include combining several individual food items from different subgroups and aggregating similar types of food items or dishes into a single FFQ item, e.g., potato dishes. Furthermore, aggregation of food items can be based on physical characteristics, e.g., savory snacks; or dietary patterns, e.g., vegetarian stews; or on eating occasions, e.g., desserts [32].

Inconsistencies exist in the literature regarding the effectiveness and accuracy of the single versus aggregated food items. The loss of information about nutrient intake can be more significant when a food group contains many food items [33]. In the present study, food items at aggregation level 1 encompass up to 23 food items at aggregation level 2 (on average, 3.5 items). For example, in the present study, the food group *wholemeal bread* (aggregation level 1) encompasses seven single food items at aggregation level 2, e.g., *wholemeal wheat bread*, *wholemeal rye* bread or *wholemeal oat bread*.

The optimized FFQs contain both the aggregated food items (aggregation level 1) and individual food items (aggregation level 2). Steinemann et al. showed that FFQs querying the less aggregated food items from one food group separately are prone to over-reporting due to the cumulative effect of including several food items. Conversely, FFQs with single food items at a higher aggregation level (e.g., eggs) may experience under-reporting as people might not report all the different ways they eat eggs, e.g., scrambled eggs or fried eggs [33].

### 4.4. Portion Sizes

For the present study, portion sizes for the created FFQs were calculated with percentiles from dietary intake data of the NVS II.

The three different scenarios show how much of the total measurement error of the FFQ is associated with different portion sizes and with the selection of food items based on MILP.

The first scenario examined the impact of different portion sizes between the 24HR and the FFQ. The mean R^2^ value in this scenario was highest, with R^2^ = 0.94, showing that portion sizes for the FFQ, derived from percentiles of the 24HR, provided accurate results for the FFQ.

The second scenario shows differences between the 24HR and the FFQ based on selected food items with MILP. Since nutrient variance constraints (see formula 3) aimed to model R^2^, the calculated R^2^ values ranged, as expected, around 0.8, with a mean R^2^ value of 0.86.

The third scenario combined the loss of information from both previous scenarios, yielding the lowest average R^2^ values with a mean of 0.81. However, this scenario represents the most realistic case as it simulates both the loss of information due to food items not included and that caused by portion sizes in the FFQ. While R^2^ was used as the primary performance metric in the present study, it does not capture systematic bias or mean-level differences between FFQ and 24HR. However, as the present study focuses on the methodological performance of the optimization framework rather than full FFQ validation, R^2^ was considered the most appropriate metric for the scenarios examined. Furthermore, as no independent validation sample was available, the reported R^2^ values should be interpreted as measures of internal consistency rather than external validity.

In the present study, portion sizes were added to the FFQs as they impact the accuracy of an FFQ.

While standard portions simplify data collection and analysis, they may systematically underestimate the nutrient intake of persons who consume large portion sizes [34,35]. Especially for foods with a large variation in portion size, categorical portion sizes (e.g., S, M, L) can be useful to increase the accuracy of nutrient intake [36].

## 5. Conclusions

The present paper introduces a framework using MILP to enhance the reliability, efficiency and effectiveness of compiling food lists for FFQs. Aggregation of food items, facilitated by MILP, was an important factor in shortening the food list. Comparisons of different FFQs against 24HR show that FFQs with selected food items from MILP and categorized portion sizes derived from percentiles of 24HR demonstrate good explanatory power for nutrient intake. MILP offers a reliable and data-driven framework for compiling optimized FFQs, which can readily be applied to more recent dietary intake data.

## Figures and Tables

**Table 1:** Number of food items included in the FFQ for food items at aggregation levels 1 and 2, calculated with mixed-integer linear programming (MILP) for energy intake (kcal) and all 41 nutrients.

	b * = 0.80	b = 0.85	b = 0.90	b = 0.95
Energy intake (kcal)				
Total number of food items	44	50	60	76
Number of food items at aggregation level 1 ** (% ***)	41 (93.2%)	47 (94%)	55 (91.7%)	67 (88.2%)
Number of food items at aggregation level 2 ** (% ***)	3 (6.8%)	3 (6%)	5 (8.3%)	9 (11.8%)
41 nutrients				
Total number of food items	66	75	87	105
Number of food items at aggregation level 1 ** (% ***)	50 (75.8%)	59 (78.7%)	65 (74.7%)	79 (75.2%)
Number of food items at aggregation level 2 ** (% ***)	16 (24.2%)	16 (21.3%)	22 (25.3%)	26 (24.8%)

* thresholds *b* indicate the percentage coverage of the first and second sets of constraints in the MILP model; ** food items were aggregated in two different levels (level 1 and 2); optimization with MILP selected the appropriate aggregation level for a food item; *** percentage from the total number of food items.

**Table 2:** Number of food items included in the FFQ for food items at aggregation level 2, calculated with mixed-integer linear programming (MILP) for energy intake (kcal) and all 41 nutrients.

	b * = 0.80	b = 0.85	b = 0.90	b = 0.95
Energy intake (kcal)				
Total number of food items	114	136	167	223
41 nutrients				
Total number of food items	140	163	195	248

* thresholds *b* indicate the percentage coverage of the first and second sets of constraints in the MILP model.

## Data Availability

The data presented in this study are available upon request from the corresponding author due to privacy reasons.

## Abbreviations

The following abbreviations are used in this manuscript:

FFQ	Food Frequency Questionnaire
24HR	24 h recall
MILP	Mixed-Integer Linear Programming
NVS II	Second German National Nutrition Survey

## Appendix A

**Table A1:** The 41 nutrients included in the optimization with MILP.

Nutrients
Energy (kcal)
Carbohydrates
Protein
Fat
SFA
MUFA
PUFA
n6 PUFA
n3 PUFA
Cholesterol
Starch
Total sugars
Glucose
Galactose
Fructose
Sucrose
Maltose
Lactose
Fibre
Alcohol
Sodium
Potassium
Calcium
Magnesium
Phosphorus
Iron
Zinc
Iodine
Retinol equivalent
Carotene
Thiamin
Niacin equivalent
Pantothenate
Riboflavin
Folate
Vitamin B6
Vitamin B12
Biotin
Vitamin C
Vitamin D
Vitamin E

**Table A2:** R^2^ * for three different scenarios to compose the FFQ and for 41 nutrients with threshold *b* = 0.8.

Nutrients	Scenario 1: FFQ with a) All Food Items that were Consumed in the 24HR and b) Calculated Portion Sizes (Percentiles from the 24HR) [95% CI] **	Scenario 2: Optimized FFQ with a) Optimized Number of Food Items and b) Original Portion Sizes from the 24HR) [95% CI]	Scenario 3: Optimized FFQ with a) Optimized Number of Food Items and b) Calculated Portion Sizes (Percentiles from the 24HR) [95% CI]
Energy (kcal)	0.94 [0.90;0.96]	0.84 [0.76;0.89]	0.78 [0.69;0.84]
Carbohydrates	0.91 [0.86;0.94]	0.80 [0.73;0.87]	0.72 [0.63;0.80]
Protein	0.96 [0.93;0.97]	0.82 [0.74;0.89]	0.79 [0.69;0.86]
Fat	0.96 [0.94;0.97]	0.85 [0.77;0.90]	0.81 [0.74;0.87]
SFA	0.96 [0.94;0.97]	0.84 [0.76;0.89]	0.81 [0.73;0.87]
MUFA	0.95 [0.93;0.97]	0.87 [0.79;0.92]	0.82 [0.74;0.87]
PUFA	0.95 [0.93;0.97]	0.86 [0.78;0.91]	0.82 [0.72;0.89]
n6 PUFA	0.95 [0.92;0.97]	0.86 [0.77;0.91]	0.81 [0.72;0.88]
n3 PUFA	0.98 [0.96;0.99]	0.88 [0.62;0.96]	0.86 [0.55;0.96]
Cholesterol	0.97 [0.95;0.98]	0.92 [0.87;0.95]	0.88 [0.83;0.92]
Starch	0.93 [0.91;0.95]	0.78 [0.70;0.85]	0.72 [0.63;0.80]
Total sugars	0.90 [0.83;0.95]	0.80 [0.69;0.89]	0.71 [0.61;0.81]
Glucose	0.91 [0.86;0.97]	0.77 [0.65;0.88]	0.69 [0.59;0.78]
Galactose	0.96 [0.95;0.98]	0.79 [0.61;0.92]	0.76 [0.58;0.89]
Fructose	0.91 [0.85;0.96]	0.90 [0.84;0.94]	0.81 [0.74;0.87]
Sucrose	0.90 [0.84;0.95]	0.78 [0.61;0.89]	0.70 [0.56;0.82]
Maltose	0.96 [0.93;0.98]	0.98 [0.95;0.99]	0.93 [0.88;0.96]
Lactose	0.95 [0.92;0.98]	0.91 [0.87;0.94]	0.85 [0.81;0.90]
Fibre	0.95 [0.92;0.98]	0.91 [0.87;0.94]	0.87 [0.83;0.91]
Alcohol	0.91 [0.87;0.97]	0.76 [0.51;0.88]	0.60 [0.38;0.70]
Sodium	0.94 [0.92;0.97]	0.89 [0.81;0.93]	0.84 [0.76;0.90]
Potassium	0.90 [0.84;0.95]	0.91 [0.87;0.94]	0.81 [0.75;0.88]
Calcium	0.93 [0.91;0.95]	0.77 [0.64;0.82]	0.70 [0.60;0.79]
Magnesium	0.92 [0.88;0.95]	0.88 [0.82;0.92]	0.83 [0.75;0.89]
Phosphorus	0.96 [0.94;0.97]	0.87 [0.80;0.91]	0.84 [0.77;0.89]
Iron	0.95 [0.93;0.97]	0.82 [0.72;0.90]	0.79 [0.67;0.88]
Zinc	0.95 [0.92;0.97]	0.78 [0.70;0.85]	0.76 [0.67;0.83]
Iodine	0.96 [0.92;0.98]	0.79 [0.62;0.89]	0.77 [0.61;0.87]
Retinol equivalent	0.93 [0.91;0.97]	0.97 [0.92;0.99]	0.90 [0.85;0.93]
Carotene	0.93 [0.91;0.97]	0.98 [0.93;0.99]	0.90 [0.85;0.94]
Thiamin	0.96 [0.93;0.98]	0.95 [0.90;0.97]	0.91 [0.84;0.95]
Niacin equivalent	0.94 [0.92;0.96]	0.85 [0.78;0.91]	0.80 [0.72;0.88]
Pantothenate	0.87 [0.73;0.97]	0.92 [0.86;0.95]	0.80 [0.66;0.90]
Riboflavin	0.95 [0.93;0.97]	0.89 [0.82;0.93]	0.85 [0.78;0.90]
Folate	0.95 [0.93;0.97]	0.87 [0.82;0.92]	0.83 [0.76;0.89]
Vitamin B6	0.94 [0.88;0.97]	0.95 [0.91;0.97]	0.89 [0.81;0.94]
Vitamin B12	0.97 [0.96;0.99]	0.73 [0.59;0.84]	0.69 [0.54;0.81]
Biotin	0.96 [0.91;0.98]	0.97 [0.93;0.99]	0.94 [0.87;0.97]
Vitamin C	0.89 [0.79;0.97]	0.93 [0.87;0.96]	0.81 [0.72;0.90]
Vitamin D	0.98 [0.97;0.99]	0.87 [0.67;0.94]	0.82 [0.64;0.91]
Vitamin E	0.92 [0.84;0.96]	0.87 [0.79;0.93]	0.80 [0.71;0.88]

* dependent variable: nutrient intake of 24HR, independent variable: nutrient intake of FFQ; ** 95% CI calculated with bootstrapping.

## References

[1] Bundesministerium für Ernährung und Landwirtschaft (2023). Ernährungsreport 2023: Deutschland, wie es isst..

[2] Blaurock J., Kaiser B., Stelzl T., Weech M., Fallaize R., Franco R.Z., Hwang F., Lovegrove J., Finglas P.M., Gedrich K. (2021). Dietary Quality in Vegetarian and Omnivorous Female Students in Germany: A Retrospective Study. Int. J. Environ. Res. Public Health.

[3] Cade J., Thompson R., Burley V., Warm D. (2002). Development, validation and utilisation of food-frequency questionnaires—A review. Public Health Nutr..

[4] Nanri A., Fujiwara A., Miyake H., Kashino I., Mizoue T. (2022). Development, Relative Validity, and Reproducibility of a Short Food Frequency Questionnaire for the Japanese. Nutrients.

[5] Nöthlings U., Hoffmann K., Bergmann M.M., Boeing H. (2007). Fitting portion sizes in a self-administered food frequency questionnaire. J. Nutr..

[6] Pérez Rodrigo C., Aranceta J., Salvador G., Varela-Moreiras G. (2015). Food frequency questionnaires. Nutr. Hosp..

[7] McNutt S., Zimmerman T.P., Hull S.G. (2008). Development of food composition databases for food frequency questionnaires (FFQ). J. Food Compos. Anal..

[8] Avital K., Tepper S., Ben-Avraham S., Shahar D.R. (2024). Development and validation of the MY-VEG-FFQ: A modular web-based food-frequency questionnaire for vegetarians and vegans. PLoS ONE.

[9] Kwiatkowska I., Olszak J., Formanowicz P., Formanowicz D. (2022). Nutritional Status and Habits among People on Vegan, Lacto/Ovo-Vegetarian, Pescatarian and Traditional Diets. Nutrients.

[10] Frisch A., Toeller M., Müller-Wieland D. (2010). Ernährungserhebungsmethoden in der Ernährungsepidemiologie. Diabetol. Und Stoffwechs..

[11] Pennington J.A.T., Stumbo P.J., Murphy S.P., McNutt S.W., Eldridge A.L., McCabe-Sellers B.J., Chenard C.A. (2007). Food composition data: The foundation of dietetic practice and research. J. Am. Diet. Assoc..

[12] Gazan R., Vieux F., Darmon N., Maillot M. (2017). Structural Validation of a French Food Frequency Questionnaire of 94 Items. Front. Nutr..

[13] Gerdessen J.C., Souverein O.W., van ‘t Veer P., de Vries J.H. (2015). Optimising the selection of food items for FFQs using Mixed Integer Linear Programming. Public Health Nutr..

[14] Blaurock J., Heuer T., Gedrich K. (2023). Optimization of a Food List for Food Frequency Questionnaires Using Mixed Integer Linear Programming: A Proof of Concept Based on Data from the Second German National Nutrition Survey. Nutrients.

[15] Heuer T., Krems C., Moon K., Brombach C., Hoffmann I. (2015). Food consumption of adults in Germany: Results of the German National Nutrition Survey II based on diet history interviews. Br. J. Nutr..

[16] Crispim S.P., Nicolas G., Casagrande C., Knaze V., Illner A.-K., Huybrechts I., Slimani N. (2014). Quality assurance of the international computerised 24 h dietary recall method (EPIC-Soft). Br. J. Nutr..

[17] Eisinger-Watzl M., Straßburg A., Ramünke J., Krems C., Heuer T., Hoffmann I. (2015). Comparison of two dietary assessment methods by food consumption: Results of the German National Nutrition Survey II. Eur. J. Nutr..

[18] Hartmann B.M., Heuer T., Hoffmann I. (2015). The German Nutrient Database: Effect of different versions on the calculated energy and nutrient intake of the German population. J. Food Compos. Anal..

[19] Dehne L.I., Klemm C., Henseler G., Hermann-Kunz E. (1999). The German Food Code and Nutrient Data Base (BLS II.2). Eur. J. Epidemiol..

[20] Wood M. (2004). Statistical Inference using Bootstrap Confidence Intervals. Significance.

[21] Ohtani K. (2000). Bootstrapping R^2^ and adjusted R^2^ in regression analysis. Econ. Model..

[22] Kurek M.A., Onopiuk A., Pogorzelska-Nowicka E., Szpicer A., Zalewska M., Półtorak A. (2022). Novel Protein Sources for Applications in Meat-Alternative Products-Insight and Challenges. Foods.

[23] Hu F.B. (2002). Dietary pattern analysis: A new direction in nutritional epidemiology. Curr. Opin. Lipidol..

[24] Subar A.F., Midthune D., Kulldorff M., Brown C.C., Thompson F.E., Kipnis V., Schatzkin A. (2000). Evaluation of alternative approaches to assign nutrient values to food groups in food frequency questionnaires. Am. J. Epidemiol..

[25] Tabacchi G., Filippi A.R., Amodio E., Jemni M., Bianco A., Firenze A., Mammina C. (2016). A meta-analysis of the validity of FFQ targeted to adolescents. Public Health Nutr..

[26] Molag M.L., de Vries J.H.M., Duif N., Ocké M.C., Dagnelie P.C., Goldbohm R.A., van’t Veer P. (2010). Selecting informative food items for compiling food-frequency questionnaires: Comparison of procedures. Br. J. Nutr..

[27] Shai I., Shahar D.R., Vardi H., Fraser D. (2004). Selection of food items for inclusion in a newly developed food-frequency questionnaire. Public Health Nutr..

[28] Eussen S.J., van Dongen M.C., Wijckmans N.E., Meijboom S., am Brants H., de Vries J.H., Bueno-de-Mesquita H.B., Geelen A., Sluik D., Feskens E.J. (2018). A national FFQ for the Netherlands (the FFQ-NL1.0): Development and compatibility with existing Dutch FFQs. Public Health Nutr..

[29] Scott Stryker W., Salvini S., Stampfer M.J., Sampson L., Colditz G.A., Willett W.C. (1991). Contributions of specific foods to absolute intake and between-person variation of nutrient consumption. J. Am. Diet. Assoc..

[30] Thompson F.E., Subar A.F., Brown C.C., Smith A.F., Sharbaugh C.O., Jobe J.B., Mittl B., Gibson J.T., Ziegler R.G. (2002). Cognitive research enhances accuracy of food frequency questionnaire reports: Results of an experimental validation study. J. Am. Diet. Assoc..

[31] Dehne L.I., Klemm C., Henseler G., Bögl K.W., Hermann-Kunz E. (1997). Der Bundeslebensmittelschlüssel (BLS II.2). Bundesgesundhbl.

[32] Ross B.H., Murphy G.L. (1999). Food for thought: Cross-classification and category organization in a complex real-world domain. Cogn. Psychol..

[33] Steinemann N., Grize L., Ziesemer K., Kauf P., Probst-Hensch N., Brombach C. (2017). Relative validation of a food frequency questionnaire to estimate food intake in an adult population. Food Nutr. Res..

[34] Køster-Rasmussen R., Siersma V., Halldorsson T.I., de Fine Olivarius N., Henriksen J.E., Heitmann B.L. (2015). Missing portion sizes in FFQ--alternatives to use of standard portions. Public Health Nutr..

[35] Welten D.C., Carpenter R.A., McPherson R.S., Brodney S., Douglass D., Kampert J.B., Blair S.N. (2000). Comparison of a dietary record using reported portion size versus standard portion size for assessing nutrient intake. Public Health Nutr..

[36] Kim M.K., Youl Choi B. (2002). The influence of portion size data on the agreement of classification of individuals according to nutrient estimates by food frequency questionnaire in a rural area of Korea. Nutr. Res..

